# Expression, purification and characterization of α-synuclein fibrillar specific scFv from inclusion bodies

**DOI:** 10.1371/journal.pone.0241773

**Published:** 2020-11-06

**Authors:** Vijay Gupta, Indulekha P. Sudhakaran, Zeyaul Islam, Nishant N. Vaikath, Issam Hmila, Tamas Lukacsovich, Prasanna R. Kolatkar, Omar M. A. El-Agnaf

**Affiliations:** 1 Neurological Disorder Research Center, Qatar Biomedical Research Institute (QBRI), Hamad Bin Khalifa University (HBKU), Qatar Foundation, Doha, Qatar; 2 Diabetes Center, Qatar Biomedical Research Institute (QBRI), Hamad Bin Khalifa University (HBKU), Qatar Foundation, Doha, Qatar; 3 Brain Research Institute, University of Zürich, Zürich, Switzerland; Tsinghua University School of Life Sciences, CHINA

## Abstract

Aggregation of α-synuclein (α-syn) has been implicated in multiple neurodegenerative disorders including Parkinson’s disease (PD), dementia with Lewy bodies (DLB) and multiple system atrophy (MSA), collectively grouped as synucleinopathies. Recently, recombinant antibody fragments (Fab, scFvs and diabodies) against α-syn have emerged as an alternative to the traditional full-length antibody in immunotherapeutic approaches owing to their advantages including smaller size and higher stability, specificity and affinity. However, most of the recombinant antibody fragments tend to be expressed as inclusion bodies (IBs) making its purification extremely challenging. In the current study, a single-chain variable fragment (scFv-F) antibody, targeting the pathogenic α-syn fibrils, was engineered and expressed in E. coli. Majority of the expressed scFv-F accumulated in insoluble aggregates as IBs. A variety of mild and harsh solubilizing conditions were tested to solubilize IBs containing scFv-F to obtain the active protein. To preserve secondary structure and bioactivity, a mild solubilizing protocol involving 100 mM Tris, pH 12.5 with 2 M urea was chosen to dissolve IBs. Slow on-column refolding method was employed to subsequently remove urea and obtain active scFv-F. A three-dimensional (3D) model was built using homology modeling and subjected to molecular docking with the known α-syn structure. Structural alignment was performed to delineate the potential binding pocket. The scFv-F thus purified demonstrated high specificity towards α-syn fibrils compared to monomers. Molecular modeling studies suggest that scFv-F shares the same structural topology with other known scFvs. We present evidence through structural docking and alignment that scFv-F binds to α-syn C-terminal region. In conclusion, mild solubilization followed by slow on-column refolding can be utilized as a generalized and efficient method for hard to purify disease relevant insoluble proteins and/or antibody molecules from IBs.

## Introduction

Synucleinopathies are a collection of neurodegenerative disorders including Parkinson’s disease (PD), dementia with Lewy bodies (DLB), and multiple system atrophy (MSA) [1]. Pathologically, these disorders are characterized by the presence of Lewy bodies that are formed by abnormal aggregation of alpha-synuclein (α-syn) protein leading to gradual degeneration of distinct neuronal population [2]. It is of utmost importance to develop reagents that can detect, bind and inhibit the toxic pathogenic species of α-syn and ultimately which can be used as potential diagnostic and therapeutic tools for synucleinopathies. Methodologies using passive immunizations utilizing conventional α-syn antibodies have been shown to improve the symptoms of PD [3, 4]. However, there are many drawbacks for such strategies including (1) the large size of monoclonal antibodies, (2) requirement of large doses for effective treatment and (3) their inability to cross the blood-brain barrier (BBB) [5].

Using recombinant DNA technology, the variable heavy chain (V_H_) and variable light chain (V_L_) sequences from an already known monoclonal antibody can be engineered into a single chain variable fragment (scFv) format. Compared to traditional antibody, scFv (consisting of V_H_ and V_L_ regions linked by a short flexible peptide linker)_._ is the smallest yet complete component that can bind to target antigen [5]. There are multiple advantages that scFv format has over the full-length antibodies (~150kDa) including smaller size (~25kDa), better tissue penetration, shorter half-life and rapid clearance, and reduced immune response due to the lack of Fc region [3, 5–9].

Expression of eukaryotic proteins in the bacterial host cells like *E*. *coli* is one of the most preferred methods to produce recombinant proteins in large quantities. However, overexpression of heterologous protein in *E*.*coli* frequently results in accumulation of partially folded target protein in the form of water-insoluble intracellular aggregates known as inclusion bodies (IBs) [10–15]. Typically isolation of recombinant protein from IBs consist of four following steps: recovery of IBs from *E*.*coli* cells by cell-lysis, solubilization of IBs, refolding of dissolved protein and purification of refolded protein [13]. Among these, solubilization and refolding of the expressed protein are the most critical steps determining the final recovery and activity of protein. Generally, IB solubilization is carried out using chaotropic reagents (urea and/or guanidine hydrochloride) at very high concentrations (6-8M) followed by removal of chaotropic denaturants by dialysis and simultaneous protein refolding methods [12, 13].

It has been reported that proteins in the IBs can exist in their native state with intact secondary structures, and mild solubilization conditions can lead to purification of the native protein [15]. Growing *E*.*coli* cultures at low temperature of 25°C has also been shown to produce non-classical IBs yielding higher percentage of active folded protein [10]. Use of mild solubilization buffers has shown to protect the protein from harsh denaturation conditions leading to the recovery of higher bioactive protein. A variety of mild solubilzation methods have been attempted involving reduced concentration of urea, adding a cycle of freeze thaw at -20°C along with low concentration of urea, dissolving urea in n-propanol, and using Tris (pH 12.5) buffered urea solution [11–14]. Solubilization of IBs has also been tried by heating in microwave in presence of 20 mM SDS [16]. All these methods have shown limited success for certain types of proteins.

In the current study we used the sequence from a well-characterized α-syn fibril-specific monoclonal antibody (Syn-F2), and engineered scFv antibody, scFv-F [4, 17]. Recombinant expression of scFv-F led to the formation of IBs, which were solubilized using a variety of mild solubilization methods. Mild solubilization method of Tris buffer pH 12.5 with 2 M urea was used to dissolve IBs containing scFv-F. Refolding of the denatured scFv-F was carried out using slow on-column buffer exchange method and finally scFv-F was purified using affinity chromatography. We determined the bioactivity of scFv-F by analyzing its binding with α-syn monomers and fibrils. We have also described structural details of scFv-F by homology modeling. Molecular docking and structural alignment studies revealed the interaction of scFv-F with residues in the C-terminal region of α-syn. The results described here demonstrate the successful expression and purification of a functional scFv having specific activity towards α-syn fibrils that can be used as potential diagnostic or therapeutic tool.

## Materials and methods

### Cloning E. coli expression construct

To engineer scFv-F construct in pET22b(+) vector, sequences of the V_L_ and V_H_ regions from mouse monoclonal antibody Syn-F2 were obtained from GenScript antibody sequencing service. The expression construct consisted of the V_L_ and V_H_ regions linked by a (Gly_4_Ser)_3_ linker and a C-terminal 6x-His tag for ease of purification was added. To clone E. coli expression-vectors, forward (32bp) and reverse primers (30bp) were designed according to the V_L_ and V_H_ sequence obtained from Syn-F2 respectively. scFv-F-pET22b(+) vector containing the coding sequence was used as a template and with the help of the newly designed forward and reverse primers, PCR was carried out to amplify scFv total coding sequence and cloned into pET28a vector. Resultant final scFv-F- pET28a construct identity was confirmed by DNA sequencing.

### Expression and purification of scFvs

For protein expression, scFv-F (pET28a) vector was transformed into chemically competent *E*. *coli* BL21(DE3) cells by heat shock. E. coli cells having scFv-F plasmid were induced for protein expression by adding 0.1 mM or 0.5 mM isopropyl β-D-1-thiogalactopyranoside (IPTG), and the cultures were induced overnight at different temperatures such as 16°C, 25°C and 37°C. The obtained culture pellet was homogenize in lysis buffer (50 mM Tris pH 8.0, 300 mM NaCl, 10% glycerol, 1X protease inhibitor, 1 mM PMSF, 20 U/ml DNAse1, and 10 mM MgCl_2_) and sonication cycles at 30 amplitude were performed for 10 sec upto 5 min with 30 sec on ice. To purify protein from inclusion bodies (IBs), the pellet was washed 4 times with washing buffer (50 mM Tris-HCl pH 8.0, 100 mM NaCl, 1% triton X-100, 1 M urea) and once with 1X PBS. To determine the ideal solution to solubilize IBs, multiple buffers such as 2 M urea solution in n-propanol, 20 mM SDS solution, 2 M urea in 100 mM Tris-HCl, pH 12.5, urea (8 M) or guanidine chloride (6 M) solutions were tried. Finally, it was decided to dissolve obtained pure IB pellet in mild solubilization buffer (100 mM Tris-HCl, 2 M urea, pH 12.5). The solubilized IBs supernatant was purified using Ni-NTA affinity chromatography (GE Healthcare). To refold the His trap column bound-scFv-protein, a linear refolding gradient from 2 M urea to 0 M urea was run using very slow speed (0.2 ml/min) of buffer exchange (on-column refolding). The non-specific bound proteins were removed by washing extensively using 40 mM and 80 mM imidazole buffer and His-tagged protein (scFv-F) was eluted using 250 mM imidazole buffer. To remove imidazole and exchange buffer to physiologic buffer, overnight dialysis using 1xPBS was performed at 4°C. To estimate protein concentration, BCA assay was used, and small aliquots of purified protein were frozen at -80°C.

### Western blot

Protein samples were boiled with 5X SDS-Laemmli sample buffer and separated on 12% SDS-PAGE gel. Protein samples in the gel were transferred to nitrocellulose membrane and the membrane was developed using anti-6X-histidine tag mouse antibody (1:2500, Abcam) followed by incubation with HRP-conjugated IgG goat anti-mouse antibody (Thermo Scientific) (dilution-1:10,000) using SuperSignal West-Pico-Chemiluminescent Substrate. BIO-RAD ChemiDoc MP imaging system was used to image the final Blots.

### Preparation of α-syn monomers and fibrils

α-Syn, different forms and other amyloidogenic forms were prepared as described earlier [17, 18]. Briefly, to prepare α-syn fibrils, 25μM of α-syn monomer protein sample was aged in PBS for 5 days at 37°C with continuous shaking at 800rpm. The formation of α-syn fibrils was monitored by Th-S fluorescence assay. To prepare monomers, α-syn was passed through a 100kDa molecular weight cut-off (MWCO) filter to remove high molecular aggregates.

### Dot blot

To perform dot blots, nitrocellulose-membrane was spotted with denoted amount of antigen (monomeric and aggregated forms of α-syn, Abeta, tau and IAPP) dissolved in 5 μl. The spotted samples were air-dried for 30min and blocked in 5% skimmed milk (in PBST). The nitrocellulose membranes were incubated overnight at 4°C with scFv-F (5μg/mL) or respective control antibodies: Syn-1 (for α-syn, BD Biosciences, 50 ng/ml) and Syn-F2 (α-syn fibril specific, 50 ng/ml), 82E1 (for Abeta, IBL America, 50 ng/ml), 5E2 (for Tau, kindly provided by prof. DominicWalsh, Harvard Medical School, 50 ng/ml,) and R10/99 (for IAPP, Abcam, 1:2000). Following incubation, blots were washed and incubated in appropriate HRP conjugated secondary Ab for 1 hr at RT and developed with west Pico chemiluminiscent substrate and imaged in Biorad scanner.

#### Indirect enzyme-linked immunosorbent assay (ELISA)

To perform ELISA, a 96-well clear MaxiSorp plate (Nunc) was coated with 100μl of 1μg /ml (single well) of α-syn monomers and fibrils in (1X PBS) and incubated overnight at 4°C. Plates were blocked (5% milk in PBST) for one hour and 100 ng, 200 ng and 400 ng of scFv-F was dispensed in the wells and incubated for 2 hours at 37°C. The plate was incubated with Anti-6x his antibody (1:2000) and goat anti-mouse secondary antibody (1:3000) and developed with 50 μl/well of TMB substrate (KPL) for 10–30 min. The absorbance was measured at 450 nm using a Perkin-Elmer atomic absorption spectrometer.

### Protein modelling and docking (scFv-F- α-syn interaction)

Due to the lack of structure of scFv-F in protein data bank (PDB), we generated scFv-F structure model by Swiss-Model [25]. Availability of several similar structures in PDB allowed facile homology modeling. Amino acid sequence of scFv-F protein was submitted. A search for templates was initialized which suggested several potential templates. Based on the protein sequence coverage and sequence identity, single chain Fv fragment of mAb735 (PDBID: 3WBD) was selected for homology modeling and three-dimensional structural models of scFv-F was generated using Swiss Model server. The quality of the predicted model was examined by MolProbity (Ref 24), a structure validation server, which revealed the results in terms of phi, psi and Cβ deviations by generating a Ramachandran plot for the resulting model. The quaternary structure analysis was performed by QSQE—a tool for the prediction of correct quaternary structure by combination of conservation scores, structural clustering, and classical interface descriptors [19].

In order to validate the scFv-F interaction with α-syn, we performed molecular docking using HDOCK docking server [20, 21]. All the currently available structures of α-syn full length proteins exist as amyloid fibril structures. In addition, short peptide structures of α-syn were also solved in conjugation with synthetic constructs or maltose binding proteins. For docking studies, α-syn fibril structure (PDBID: 6A6B, comprising amino acids 37–99) was retrieved and manually processed to remove water and heteroatoms. Due to the large size of fibril, it was used as a receptor and scFv-F was modeled as ligand. We performed the docking with default parameters and 10 predicted conformers (poses) were further analysed. All structural analyses including figures with structure representations and superimposition were produced using the program UCSF Chimera [22].

## Results

### Engineering of α-syn fibril-specific scFv and its expression in E. coli

**Fig 1:**
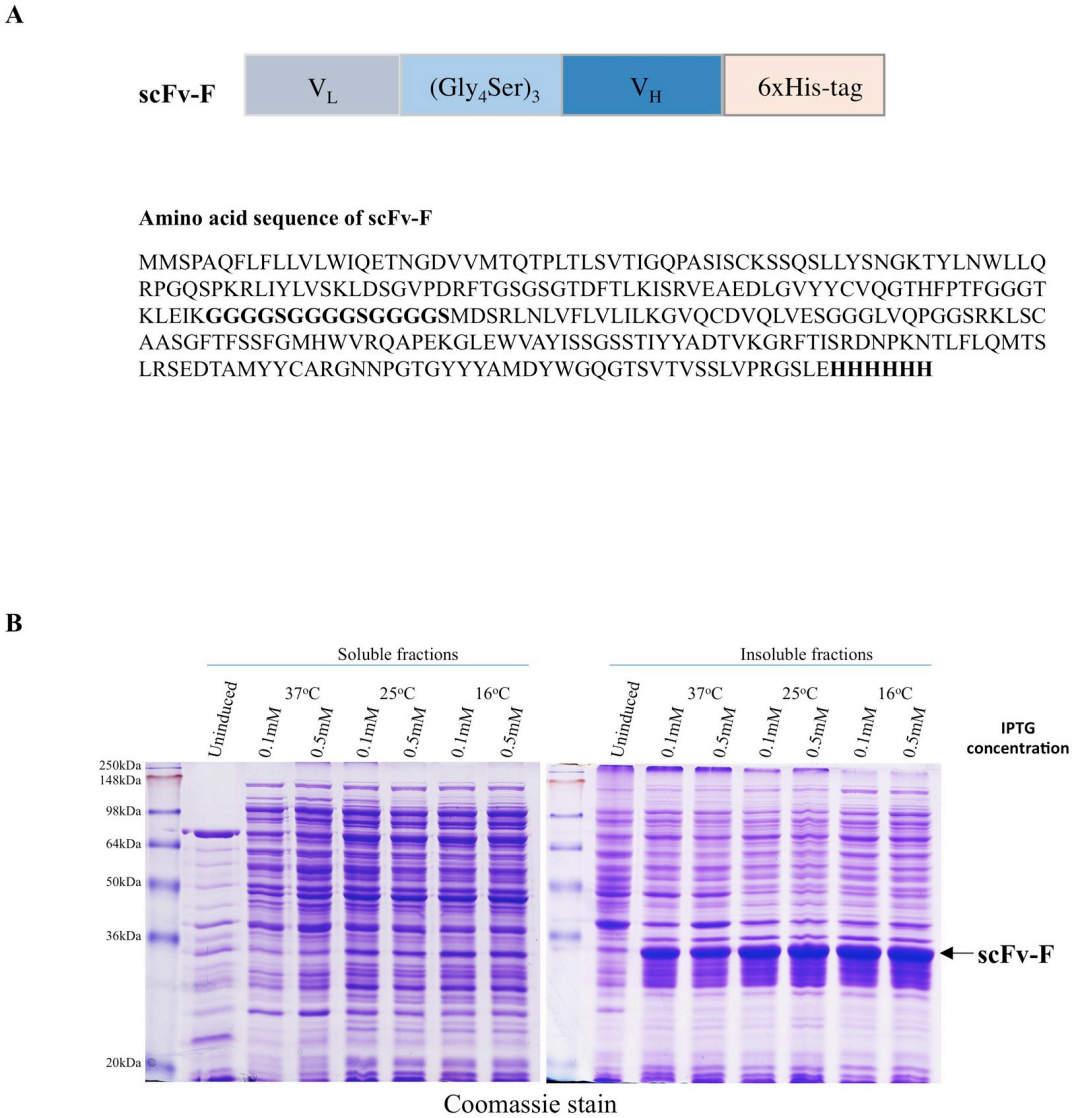
Schematic representation of scFv-F and its expression analysis. (a) To engineer scFv-F, nucleotide coding sequences for light chain (V_L_) and heavy chain (V_H_) variable domains were connected by a (Gly_4_Ser)_3_ linker with a C-terminal 6xHis-tag. Amino acid sequences translated from the coding sequence of scFv-F is presented, (Gly_4_Ser)_3_ and 6X-His tag is in bold letters. (b) Coomassie stain analysis of protein expression of scFv-F in soluble/insoluble fractions. Protein expression was induced by overnight incubation at 37°C, 25°C and 16°C with different IPTG concentrations as indicated and protein samples were run on 12% SDS-PAGE. scFv-F protein band is indicated by an *arrow*.

The single-chain variable fragment (scFv) antibody, scFv-F was generated by cloning DNA sequences of V_L_ and V_H_ domains from Syn-F2 antibody sequence in pET-28a expression vector. The variable domains were connected by a Glycine-Serine linker, (Gly_4_Ser)_3,_ sequence and a 6x-His tag at the C-terminus for convenient purification (Fig 1a). Protein expression construct for scFv-F was transformed into *E*. *coli* BL21 (DE3) chemical competent cells and induction profiling was performed using 0.1 and 0.5 mM IPTG at different temperatures. Expression profiling of the cell-lysates by SDS-PAGE showed very low expression of scFv-F in the soluble fraction (Fig 1b). Moreover, varying different parameters including temperature (16°C, 25°C, and 37°C), IPTG concentrations (0.01–1.0 mM) or addition of chemical chaperones/chaotropic agents (sorbitol, ethanol, and L-arginine), did not improve the expression of the scFv-F in the soluble fraction.

Overexpression of exogenous proteins in *E*. *coli* results in protein aggregation leading to the formation of insoluble IBs. Consistent with this, scFv-F had higher expression in the insoluble fraction compared to the soluble fractions (Fig 1b). Upon quantification, we found that scFv-F had highest expression in the insoluble fractions at 16°C with 0.1 mM IPTG (Fig 1b and S2a Fig). These newly formed IBs containing the partially folded protein may have advantages such as (a) ease of isolation from bacterial cells by cell-lysis because IBs have different size and density compared to cellular components, (b) presence of less degraded protein due to resistance from cellular protease in the closed environment of IBs and (c) higher level of protein homogeneity due to protein accumulation in IBs. However, isolating pure active protein from IBs is a challenging task involving multiple steps such as solubilization of IBs and refolding of the denatured protein.

### Comparison of inclusion bodies solubilization using different methods

As the majority of scFv-F was expressed as IBs, we decided to purify the scFv-F from the IBs. Expression of scFv-F in the insoluble fractions was found to be highest upon induction with 0.1 mM IPTG overnight at 16°C (Fig 1b and S2a Fig). IBs are conventionally solubilized using high concentrations of chaotropic agents such as urea (8M) or guanidine chloride (6M) causing complete denaturation of active protein, making it unable to exhibit its antigen-binding properties. Removal of these denaturants (urea or guanidine chloride) from solubilized proteins was achieved by a slow and sequential dialysis protocol simultaneously refolding the inactive protein to its active form. According to some reports, IBs are the reservoir of aggregated proteins where some portion is in the form of native or native-like protein with some secondary structures maintained [15]. Therefore, solubilization conditions, which do not cause complete denaturation of protein content in IBs, can be of use in purification of this native protein while maintaining bioactivity.

**Fig 2:**
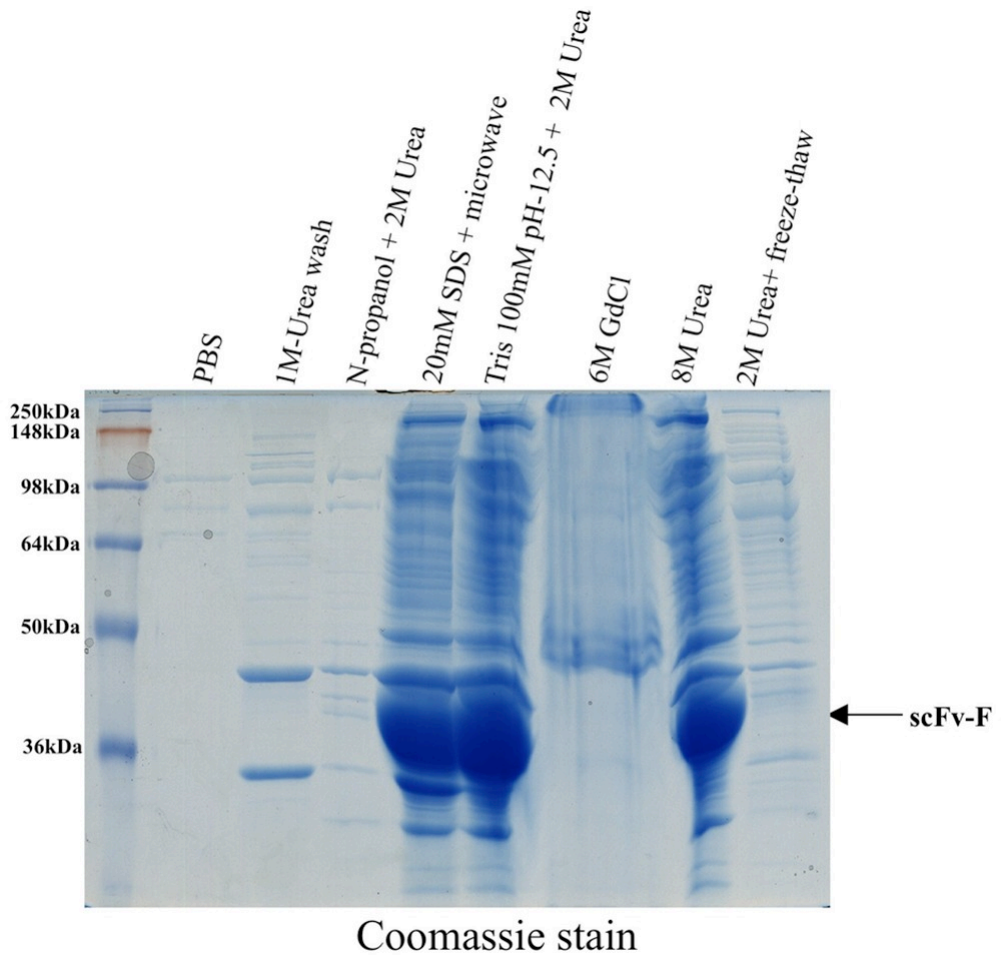
Comparison of inclusion body solubilization using different methods. Overnight protein expression (scFv-F) was induced at 16°C with 0.1 mM IPTG concentration and obtained inclusion bodies were solubilized using different buffers or conditions (1X PBS-pH 7.4 or 1 M urea wash, or solutions containing n-propanol + 2 M urea, 20 mM SDS + microwave treatment 20 sec, Tris 100 mM, pH 12.5 + 2 M urea, 6 M GdCl, 8 M urea and 2 M urea with 3 freeze thaw at -20°C). scFv-F protein band is indicated by an *arrow*.

We found that many existing protocols utilize reduced concentration of 2 M urea so that complete denaturation of proteins accumulated as IBs can be prevented. We decided to compare many such methods to find the most suitable condition to purify scFv-F. The methods we tried were 2 M urea solution with a cycle of freeze thaw at -20°C, 2 M urea solution in n-propanol, and 2 M urea dissolved in Tris pH 12.5 buffer [11–14] (Fig 2). We also tried dissolving scFv-F IBs with microwave heating in 20 mM SDS solution [16] (Fig 2). The conventional methods of IBs solubilization using urea (8M) or guanidine chloride (6M) were also tried. Though all these protocols have been shown to work for several proteins, the method involving urea solution in n-propanol and protocol involving freeze thaw cycle did not work for scFv-F (Fig 2). We obtained similar solubilization results using other methods such as microwave heating in 20 mM SDS, 100 mM Tris with 2 M urea at pH 12.5 and 8 M urea solution. IBs were isolated from IPTG induced *E*. *coli* cells by lysis using sonication, washed with 1 M urea and 1% detergent containing wash buffer (4 times) and ultimately with 1X PBS to generate purified IBs. Finally, we decided to use a mild solubilization buffer containing 100 mM Tris, and 2 M urea at pH 12.5 to solubilize the purified scFv-F-IBs. Based on the amino acid sequence analysis, we found that scFv-F has a pI of 8.72. Due to the pH difference relative to scFv-F (pI-8.72) this mild solubilization Tris buffer of pH 12.5 containing 2 M urea can solubilize scFv-F protein by breaking its ionic and hydrophobic interactions.

### On-column refolding of solubilized scFv-F and affinity purification

Recovery of purified active protein from IBs depends on two critical factors, i) the solubilization from IBs maintaining partially folded structure and ii) refolding to generate activity in the protein. Active solubilized protein can subsequently be purified using affinity chromatography. There are multiple methods for refolding solubilized denatured proteins from IBs including dialysis and dilution, however most of them involve multiple time-consuming steps with large amount of fresh buffer requirements. We chose to follow FPLC based on-column refolding protocol due to its ease and effectiveness [23].

**Fig 3:**
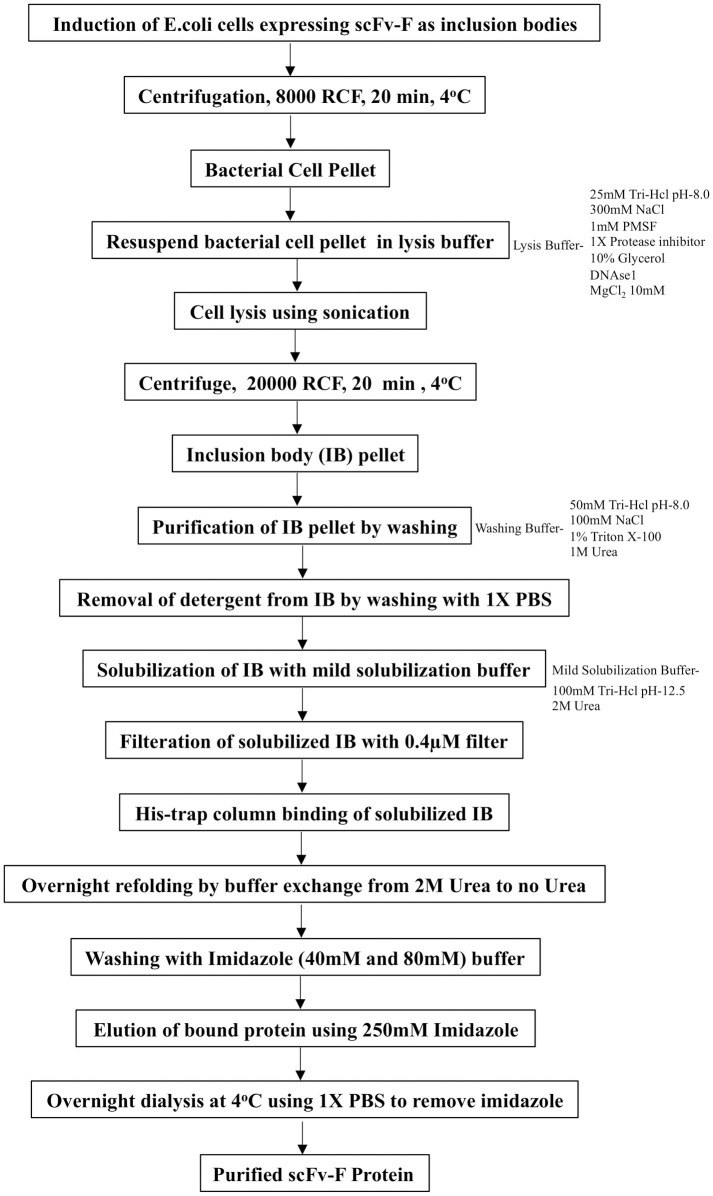
Schematic representation of the IBs isolation and solubilization followed by overnight slow refolding and affinity purification of scFv-F.

**Fig 4:**
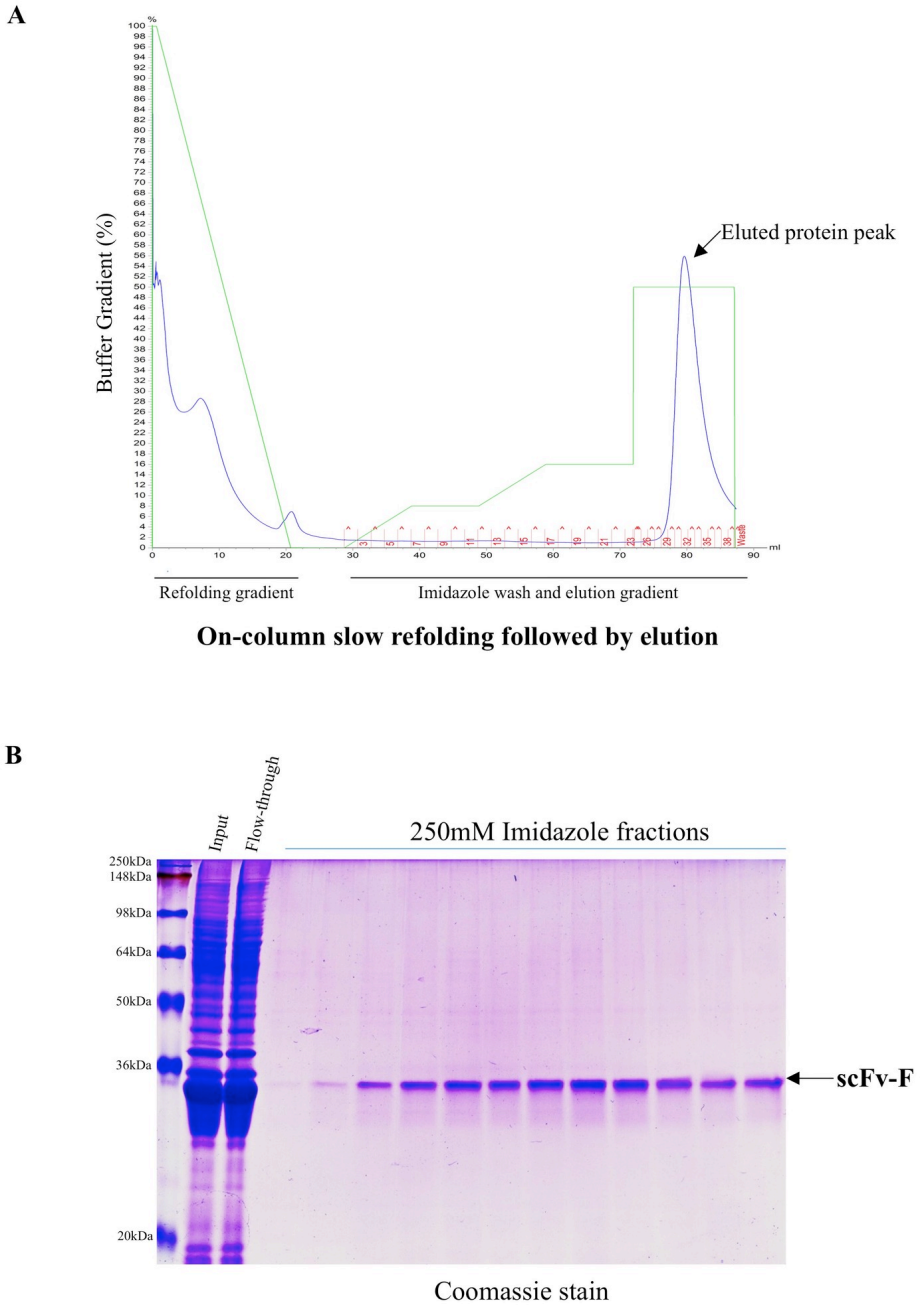
On-column slow refolding followed by elution. (a) FPLC chromatogram demonstrating the FPLC run and the eluted protein peak. Obtained inclusion body pellet was solubilized using buffer containing Tris 100 mM, pH 12.5 + 2 M urea and loaded on His-trap column and overnight slow refolding was performed by replacing buffer containing Tris 100 mM, pH 12.5 + 2 M urea with Tris 25 mM pH 8.0 + 300 mM NaCl. After overnight refolding, bound non-specific proteins were washed with two imaidazole washes (4 CV) of 40 mM and 80 mM followed by bound protein elution using 250 mM Imidazole buffer. (b) Coomassie stain analysis of protein expression of scFv-F in imidazole eluted fractions. Eluted protein samples were boiled in 1X sample loading buffer and run on 12% SDS-PAGE and after the gel run, it was stained using Coomassie stain and destained in destaining buffer. scFv-F protein band is indicated by an *arrow*.

As described in Fig 3 schematic diagram, IBs solubilized using mild solubilization buffer were filtered and loaded on His-trap column in presence of 2 M urea buffer. After passing through the column, bound denatured protein was refolded by buffer exchange from 2 M urea to buffer with no urea. This on-column buffer exchange was achieved by setting up the buffer transfer for overnight (at least for 12 hours) with very low flow rate (0.2 ml/min). Following this, column was washed with buffers containing 40 mM and 80 mM imidazole (5 CV each) to remove bound impurities and finally the bound His-tagged scFv-F was eluted using buffer with 250 mM imidazole. Chromatogram of the FPLC run showed the elution of scFv-F protein as single peak (Fig 4a). Purity of the eluted scFv-F was checked on SDS-PAGE gel and the fractions containing the pure scFv-F protein were pooled and dialysed against 1X PBS pH 7.4 (Fig 4b). The final yield obtained for the scFv-F protein was 0.5 mg/liter.

### Characterization of purified scFv-F

**Fig 5:**
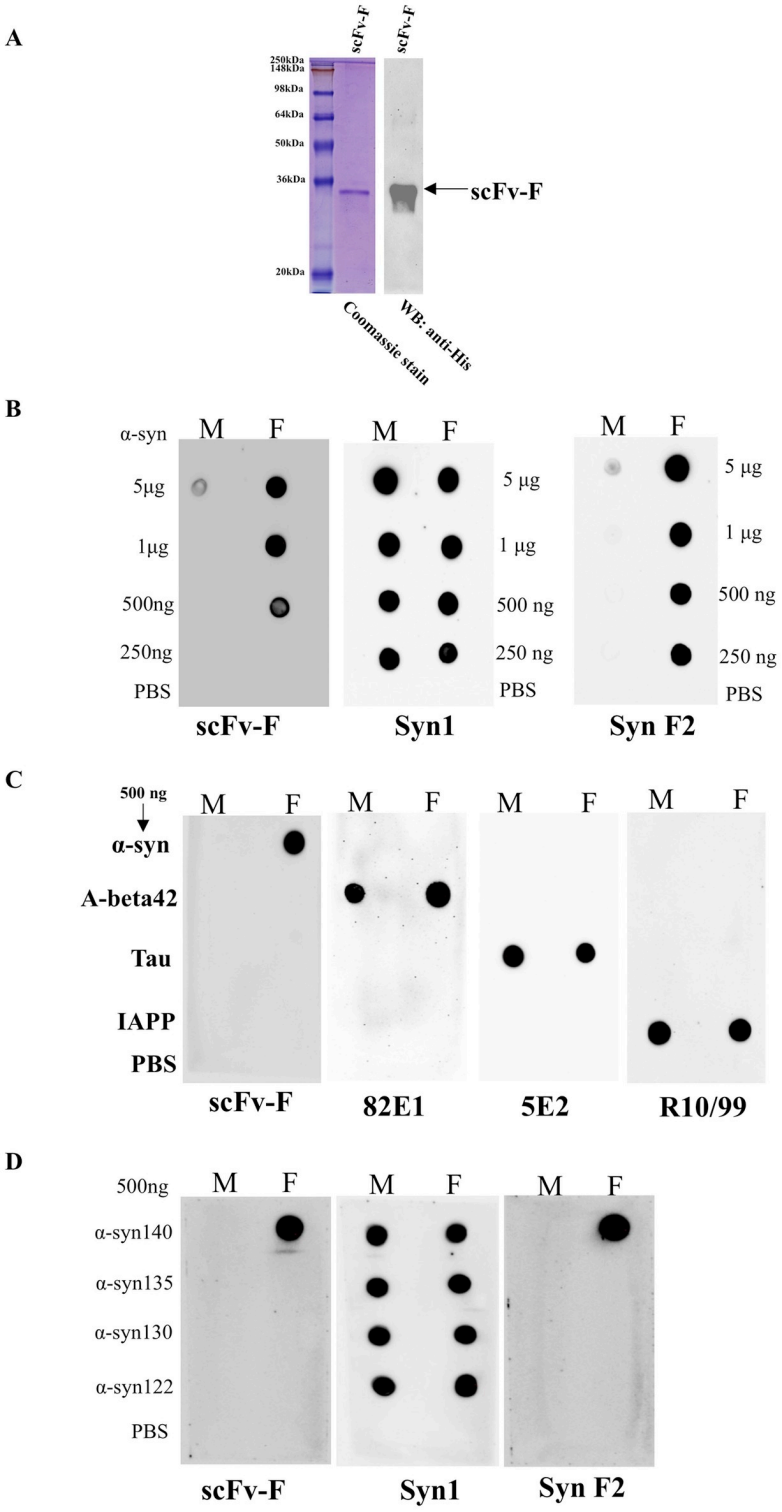
Characterization of purified scFv-F. (a) SDS-PAGE (left) and western blot (right) analysis of His-trap purified and dialysed scFv-F protein. One μg of boiled protein sample was loaded for SDS-PAGE (left) and 100 ng for western blot (right) analysis. Molecular masses (kDa) of protein standards are indicated on the left. (b) Dot blot showing specific binding of scFv-F to fibrillar form of α-syn. Indicated amounts of full-length α-syn monomers (M) and fibrils (F) were spotted onto a nitrocellulose membrane. The membranes were probed with scFv-F followed by anti-His, Syn-F2 and Syn-1 to detect full-length α-syn. (c) Dot blot to detect specific binding of scFv-F to fibrillar form of α-syn compared to other amyloid fibrils. Fibrillar (F) and monomeric (M) forms (1μg) of α-syn, A-beta42, Tau and IAPP were spotted onto a nitrocellulose membrane. The membranes were probed with scFv-F, 82E1 for A-beta42, 5E2 for Tau, R10/99 for IAPP antibodies. (d) Dot blot was performed to determine binding of scFv-F to various truncated forms of α-syn protein (α-syn135, α-syn130 and α-syn122) compared to the full length α-syn140 as described above. ScFv-F show specific binding with only the full-length α-syn140 fibrils but not with the truncated proteins.

Western blotting using anti-His antibody confirmed the presence of full-length scFv-F protein after purification (Fig 5a). Further we utilized dot blot to determine the specificity of the purified scFv-F protein. Purified α-syn monomers and fibrils were spotted on nitrocellulose membranes and probed using the scFv-F followed by anti-His antibody. We found that the scFv-F bound with higher specificity to α-syn fibrils compared to α-syn monomers (Fig 5b) with up to 500 ng α-syn fibrils (Fig 5b). Syn-F2 antibody was used as a positive control for α-syn fibrils and Syn-1 antibody to determine the equal loading of α-syn antigens (Fig 5b). After determining the specificity of scFv-F for α-syn fibrils, we wanted to test whether this binding is specific for α-syn fibrils or the scFv-F can non-specifically bind to any other type of amyloid proteins. Monomeric and fibrillar forms of various amyloid proteins such as Abeta42, Tau, and islet amyloid polypeptide (IAPP) were coated and dot blots were performed. ScFv-F showed binding only with α-syn fibrils and not with fibrils or monomers of other amyloid proteins, confirming its specificity towards α-syn. The A beta, Tau and IAPP samples however showed good reactivity to their own respective control antibodies 82E1, 5E2 and R10/99 respectively (Fig 5c). We also checked the requirement of C-terminal region of α-syn protein in binding with scFv-F by dot blot. We used monomers and fibrils of different truncated forms of α-syn namely α-syn135, α-syn130 and α-syn122 and compared their binding with respect to full-length α-syn140. We show that scFv-F fail to bind to α-syn fibrils when they are truncated from their C-terminal indicating the requirement of C-terminal amino-acids in binding with scFv-F (Fig 5d). To gain further insight about scFv-F activity, we performed indirect-ELISA and found that scFv-F detected α-syn140 fibrils significantly higher than α-syn140 monomers (S2b Fig). Moreover, these results also confirmed that purified scFv-F protein had correctly folded antigen-binding sites that efficiently detected the α-syn fibrils. Further characterization of scFv-F using various *in vitro* and cell-line based assays has been recently accepted for publication [24].

### Structural details of scFv-F and potential interaction with α-syn

**Fig 6:**
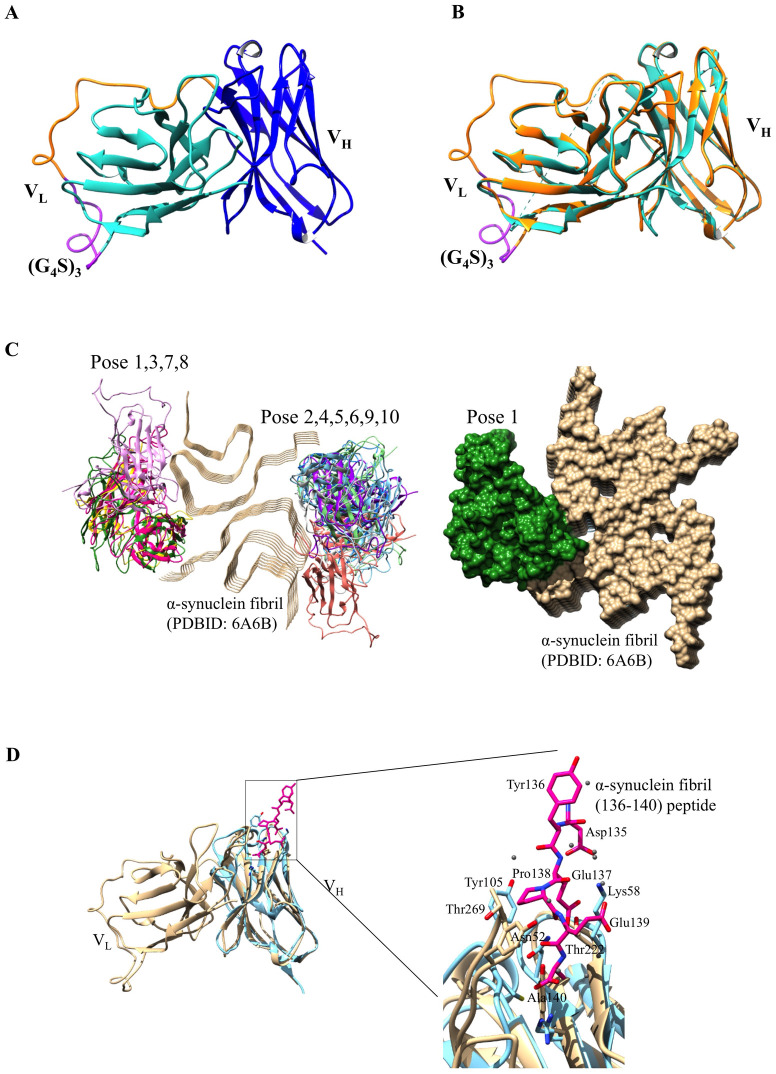
Overall structure of modelled scFv-F. (a) Cartoon representation of modelled scFv-F. The V_L_ and V_H_ domains are coloured green and blue, respectively. The disordered GS linker region is shown as purple. (b) Structural superimposition of modeled scFv-F (orange) with single chain Fv fragment of mAb735 (PDBID: 3WBD, green). The two structures are highly similar in overall fold. (c) Potential binding of modelled scFv-F to α-syn. Molecular docking of modeled scFv-F onto α-syn fibril structure (PDBID: 6A6B, golden) represented as cartoon and surface. (d) Structure superimposition of modeled scFv-F (golden) with: α-syn complex peptide (PDBID: 2X6M, cyan). Boxed zoom-in view shows binding pocket and residues involved. Dots represent water molecules and were shown to be involved in mediating the interaction.

We performed the scFv-F structure homology modelling using SWISS-MODEL [25], an automated server to generate 3D models of proteins. The submitted protein sequence retrieved several potential templates for homology modeling. We aligned the top five sequence templates (S1a Fig); the sequence identity of these templates with respect to scFv-F varies from 56 to 62%, highest for 3WBD. We selected the 3WBD as the template based on its highest global model quality estimation (GMQE) score of 0.69 where quality estimation combines properties from the target-template alignment. Swiss-Model generated the scFv-F monomeric model. The modeled structure contains V_L_ and V_H_ domains, a typical fold of the variable domains of an immunoglobulin, similar to its template and other scFvs (Fig 6a). Each domain is composed of two antiparallel β-sheets arranged in a β-sandwich fold. Structural comparison between modelled scFv-F and single chain Fv fragment of mAb735 (PDBID: 3WBD) showed that the structures are very similar, with a root mean square deviation value of 0.64 Å (Fig 6b).

Molecular docking of modeled scFv-F and α-syn was performed by HDOCK docking server [20, 21]. HDOCK is a fast protein-protein docking server that automatically predicts their interaction through a hybrid algorithm of template-based and template-free docking [20, 21]. The modeled scFv-F docks at the interface of two protofilaments (Fig 6c). Previous studies on cryo EM studies on α-syn fibrils shows that α-syn subunits in each protofilament stack along the fibril axis with a helical twist and the protofilaments intertwine along the symmetrical screw axis [26]. All the poses interacted with the fibril at the protofilament interface, which is considered as the most stable region of the fibril [26]. To investigate the potential residues involved in interaction, we aligned our modeled structure to NbSyn2:α-syn (PDBID: 2X6M). NbSyn2:α-syn is a single-domain camelid antibody complexed with residues 132–140 of α-syn (N-GYQDYEPEA-C) [27]. The structure superimposed well with a root mean square deviation value of 0.96 Å (Fig 6d), suggesting that the interactions may be conserved. A closer look at the interaction interface (Fig 6d) revealed that the peptide binds to single-domain camelid antibody in a pocket formed by residues of the complementarity-determining region (CDR) loops and makes contacts with residues Tyr136, Glu137, Pro138, Glu139, and Ala140 of α-syn [27]. By similarity, Pro138 of the α-syn peptide interacted with Thr269 of modelled scFv-F. Ala140 of peptide is deeply buried in CDR loops and interacted with Tyr215 of modelled scFv-F. Apart from the direct interaction, all these amino acids also interact directly through water molecules. Comparison also revealed that the residues of α-syn that interact with our modeled structure are bound in an extended conformation, a feature that is commonly found in antibody-peptide complexes [27, 28].

## Discussion

Antibody based therapies have turned out to be the most promising candidates towards tackling synucleinopathies [17, 29]. The large size of antibodies often makes them unable to cross the blood-brain barrier (BBB) limiting their widespread usage. With the advent of recombinant DNA technology, scFvs have been engineered by combining variable regions of heavy and light chain containing polypeptide linker to exploit already known monoclonal antibody sequences [5]. Due to their smaller size, scFvs have multiple benefits over traditional antibodies in terms of higher permeability through BBB and better efficiency as well as the ease of genetic manipulation enhancing their affinity and intracellular targeting. Moreover, scFvs can be linked with tracer chemicals generating *in vivo* imaging diagnostic tools in addition to their potential therapeutic usage [5].

Production of large amount of active and pure recombinant scFv is the prerequisite prior to its characterization and usefulness as therapeutic or diagnostic tool. Quite often, expression of scFvs in *E*. *coli* host leads to the formation of insoluble IBs. Therefore, solubilizing these IBs to produce the active folded protein is of utmost importance. Recent literature has shown that IBs contain some amount of protein with native-like secondary structures and *E*.*coli* cells grown at low temperature such as 25°C have non-classical IBs with correctly folded protein [10, 15, 30]. Successful isolation of active folded protein from classical and non-classical IBs encouraged us to purify scFv-F from IBs.

First step towards isolation of active folded protein from IBs is to solubilize insoluble protein aggregates found inside the IBs. High amount of chaotropic agents such as urea (8 M) and guanidine hydrochloride (6 M) or detergents such as sodium dodecyl sulfate (SDS) or n-cetyl trimethylammonium bromide (CTAB) are used to solubilize these IBs [12, 31]. Chaotropic agents lead to the denaturation of the proteins making them inactive whereas detergent may interfere with the downstream purification processes [12, 31]. Removing these reagents since they are inhibitory for most of protein activities as well as structural integrity and maintaining protein function is the next challenge. Mild solubilization conditions maintaining the folding status of proteins in IBs are therefore the most optimum approach. Different mild methods of IBs solubilization have been described in the literature such as 2 M urea dissolved in n-propanol (protein unfolding due to removal of hydrophobic interaction and reduction of dielectric constant by organic solvent), 100 mM Tris, pH 12.5 (high pH break hydrophobic as well as ionic interaction), 2 M urea with freeze thaw cycle (stress generation by lowering temperature to denature protein) and microwave in presence of 20 mM SDS (heat energy with detergent) solubilizing IBs while maintaining secondary structures [10–16, 30–32].

We selected the 100 mM Tris, pH 12.5 with 2 M urea buffer-based method to solubilize IBs expressing scFv-F due to its effectiveness. Next hurdle was to refold the solubilized protein while removing the urea present in solubilization buffer. Pulse renaturation and slow sequential dialysis are the two common methods to remove the denaturant used in solubilization process [12]. In pulse renaturation, a small amount of denatured protein is added to a large amount of dialysis buffer drop by drop whereas in slow sequential dialysis the high amount of denaturant is replaced by sequentially reducing the concentration until there is no denaturant left in the buffer e.g. from 2 M to 1 M to 0.5 M to 0 M urea to prevent protein aggregation. However, while refolding scFv-F, both these methods led to protein aggregation hindering our efforts to isolate active protein.

We turned our attention to solid state refolding methods wherein protein refolding is carried out in the bound state on the affinity column [23]. In this method, first protein is passed through the affinity column (His-trap) in denatured state, and after the complete binding occurs, denaturation buffer is slowly replaced by regular buffer lacking the denaturant. The process is carried out overnight in slowest possible speed to give enough time to denatured protein to refold. This slow buffer exchange not only removed the 2 M urea from the protein solution but also led to pH conversion from pH 12.5 to pH 8.0 since the new buffer passed through the column is 25 mM Tris-HCl, pH 8.0 with NaCl 300 mM. After this buffer exchange and refolding, the bound pure scFv-F protein is eluted from the affinity column using imidazole buffer.

Finally, we employed *in silico* studies to understand the structural details as well as to investigate the structural basis of scFv-F-α-syn interaction. Structural alignment of known scFv structure on homology modelled scFv-F showed a highly conserved immunoglobulin fold. The docking complex of scFv-F-α-syn was analysed and the binding pocket for the C-terminal region of α-syn peptide was predicted based on superimposition to an already solved structure. Although a thorough study on the binding of the scFv-F-α-syn is required, preferably by biophysical techniques like isothermal titration calorimetry and x-ray crystallography, our modeling and docking studies provided preliminary insights into the binding properties of scFv-F with α-syn.

The importance of this work lies in engineering and purification of single-chain variable-fragment (scFv-F) antibody, targeting the pathogenic α-syn fibrils. We have developed mild solubilizing protocol followed by slow on-column refolding to dissolve IBs and obtain active scFv-F. We show that isolated antibody fragments (scFv-F) selectively bind fibrillar α-syn on dot blots. Our findings underscore the potential value of scFvs as therapeutic of synucleinopathies. The main advantage of this method is the combination of mild denaturation with a less time consuming method of refolding compared to slow and tedious multiple dialysis steps to refold the proteins. The method might not work for pH-sensitive proteins or aggregation prone proteins during on-column refolding process.

## Conclusion

To summarize, we demonstrate a simple, useful and efficient method for solubilization of IBs using Tris-HCl, 100 mM, pH 12.5 with 2 M urea buffer followed by slow on-column refolding and affinity purification, for obtaining pure and active scFv. This method not only dissolves the IBs using mild solubilizing conditions, preserving native and secondary structures of the proteins, but also addresses refolding of denatured proteins in a standardized protocol employing on-column slow refolding. This method could be used to purify a variety of proteins in IBs at large scale in an automated manner efficiently.

## Supporting information

S1 Fig Protein sequence alignment of scFv-F. **(a)** Protein sequence alignment of scFv-F, 3wbd—single chain Fv fragment of mAb735, 3auv—sc-dsFv derived from the G6-Fab, 1jp5—single-chain Fv fragment 1696, 1ktr—Anti-his tag antibody 3d5 variable light chain, Peptide linker, Anti-his tag antibody 3d5 variable heavy chain, and 3ux9—ScFv antibody. The identical residues were shown as red, while similar residues as blue. The linker GS region was highlighted with yellow colour. Alignment provided a clear sequence similarity (close to 60%) between our query sequence and the template sequences. **(b)** Model Quality assessment using MolProbity software shows that 91.35% of residues found in the alignment fall under Ramachandran favourable region.
(TIF)

S2 Fig Quantification of expression profiling of scFv-F at different temperatures with varying IPTG induction and scFv-F activity by ELISA. **(a)** SDS-PAGE coomassie stained gels showing expression of scFv-F in insoluble fractions from three different experiments were quantified using densitometric analysis and scFv-F protein levels were plotted. Statistical analysis was performed using 2way ANOVA with Tukey’s multiple comparisons test (****, p< 0.0001). **(b)** ELISA showing specific binding of scFv-F to fibrillar form of α-syn. 100 ng of α-syn monomers or fibrils were coated on a 96-well MaxiSorp plate and indirect ELISA was performed using indicated concentrations of scFv-F. Statistical analysis was performed using 2way ANOVA with Sidak’s multiple comparisons test (****, p< 0.0001).
(TIF)

S1 File (PDF)
