# Supplementary figures and images for: Expression, purification and characterization of α-synuclein fibrillar specific scFv from inclusion bodies
Source: PLoS One. 2020 Nov 6;15(11):e0241773. doi: 10.1371/journal.pone.0241773 (PMC7647061; doi:10.1371/journal.pone.0241773)

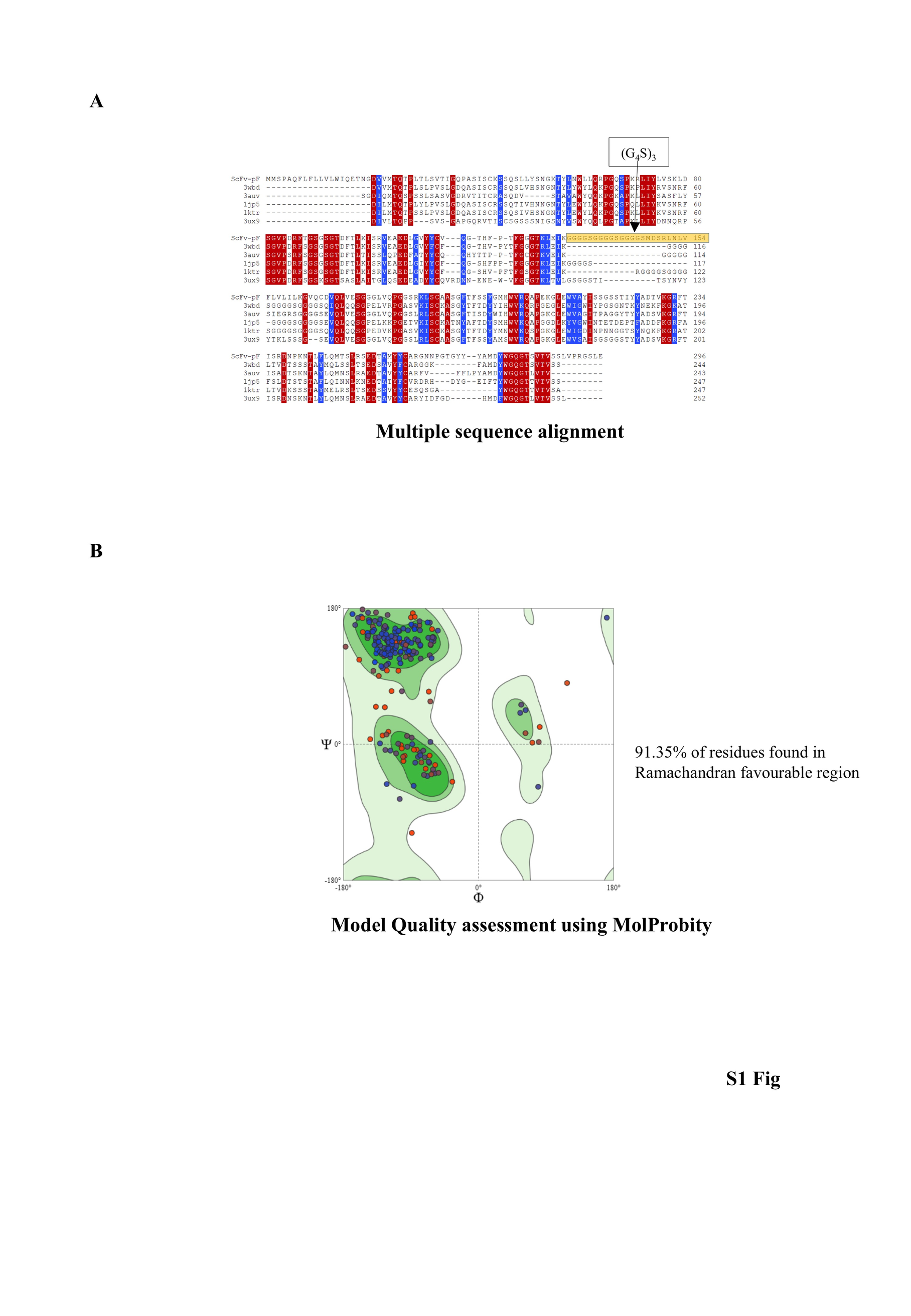

Supplement: S1 Fig — (a) Protein sequence alignment of scFv-F, 3wbd—single chain Fv fragment of mAb735, 3auv—sc-dsFv derived from the G6-Fab, 1jp5—single-chain Fv fragment 1696, 1ktr—Anti-his tag antibody 3d5 variable light chain, Peptide linker, Anti-his tag antibody 3d5 variable heavy chain, and 3ux9—ScFv antibody. The identical residues were shown as red, while similar residues as blue. The linker GS region was highlighted with yellow colour. Alignment provided a clear sequence similarity (close to 60%) between our query sequence and the template sequences. (b) Model Quality assessment using MolProbity software shows that 91.35% of residues found in the alignment fall under Ramachandran favourable region. (TIF) [file pone.0241773.s001.tif]

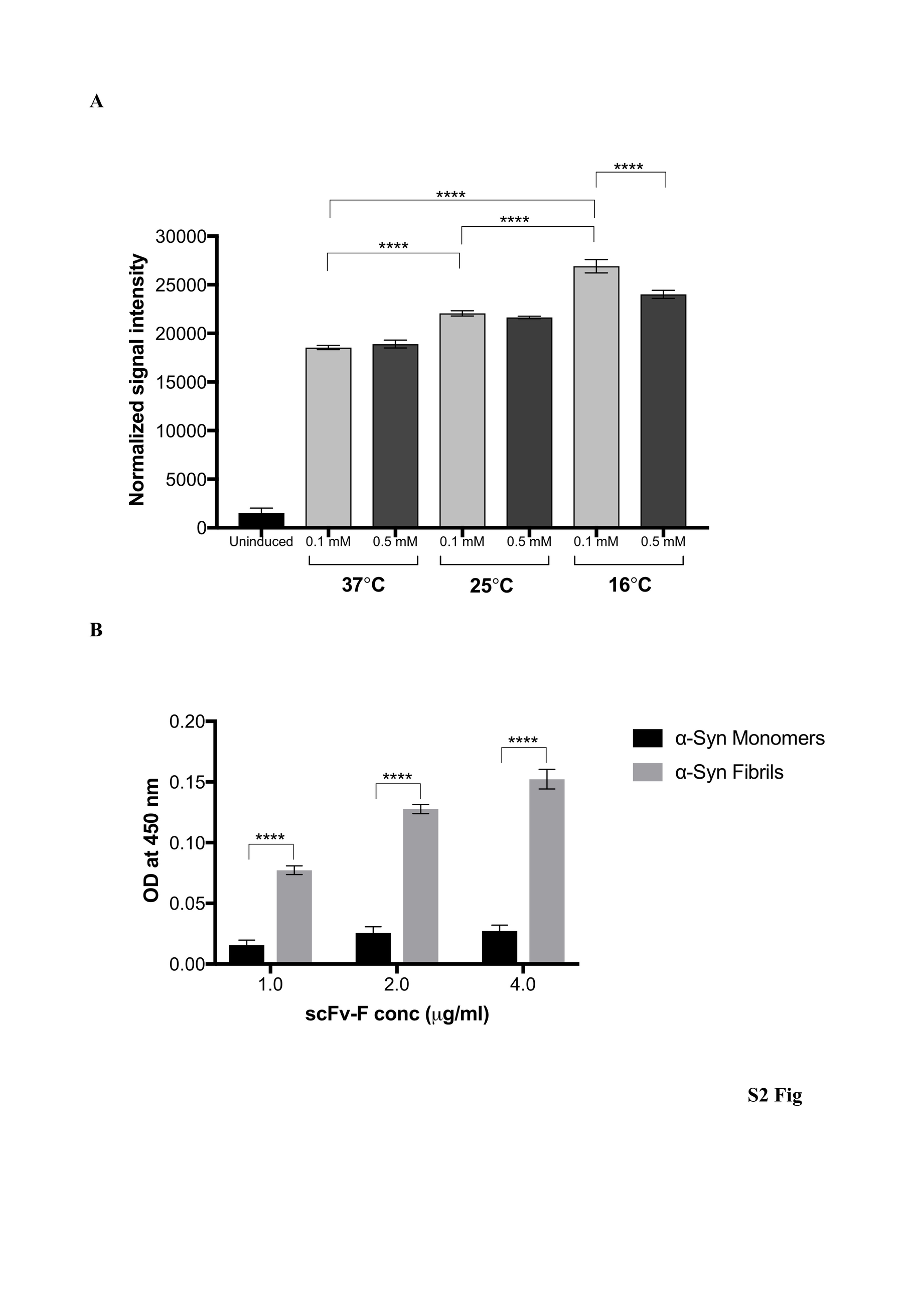

Supplement: S2 Fig — (a) SDS-PAGE coomassie stained gels showing expression of scFv-F in insoluble fractions from three different experiments were quantified using densitometric analysis and scFv-F protein levels were plotted. Statistical analysis was performed using 2way ANOVA with Tukey’s multiple comparisons test (****, p< 0.0001). (b) ELISA showing specific binding of scFv-F to fibrillar form of α-syn. 100 ng of α-syn monomers or fibrils were coated on a 96-well MaxiSorp plate and indirect ELISA was performed using indicated concentrations of scFv-F. Statistical analysis was performed using 2way ANOVA with Sidak’s multiple comparisons test (****, p< 0.0001). (TIF) [file pone.0241773.s002.tif]

B

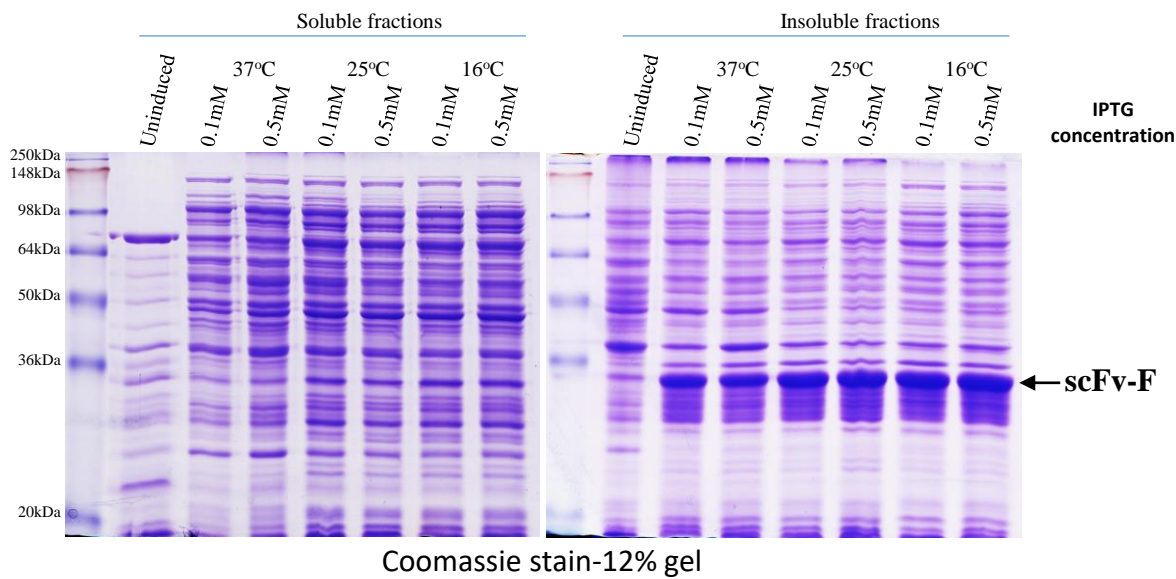

Figure-1

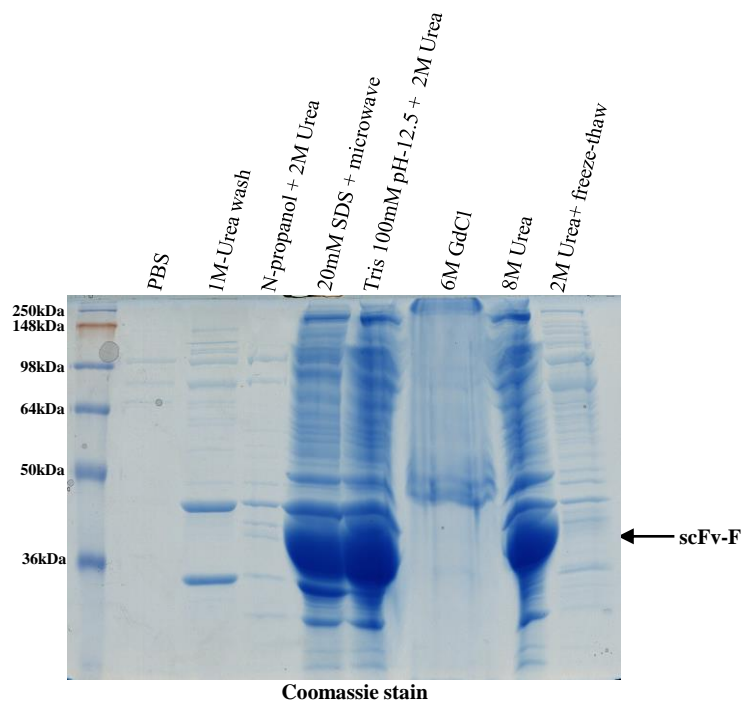

**Figure-2**

**B**

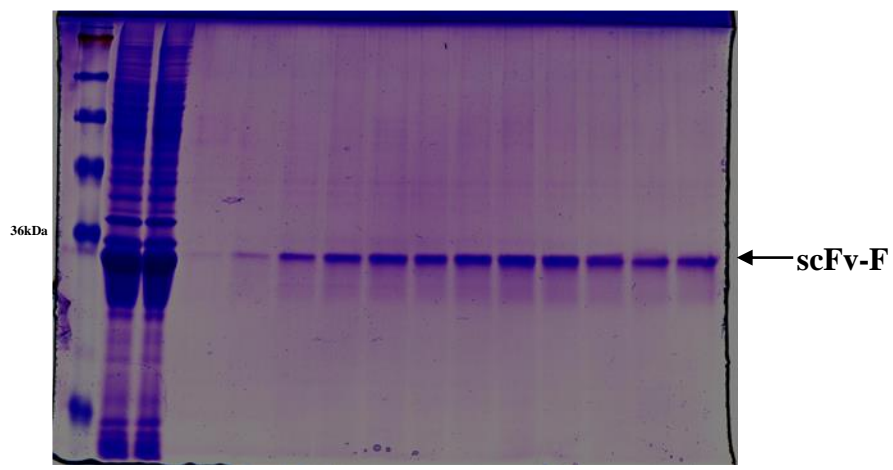

**Figure-3**

A

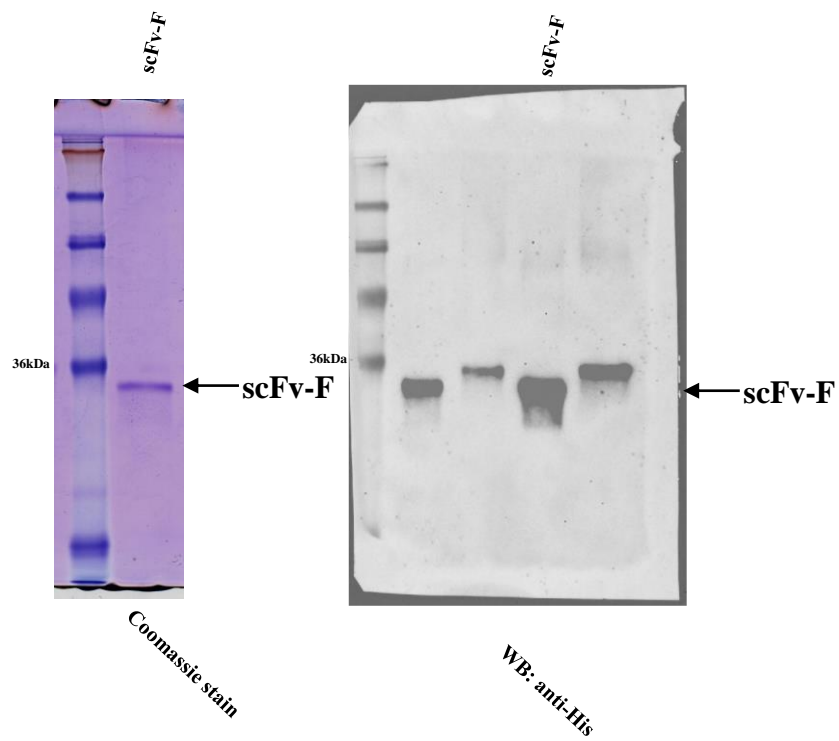

Figure-5

Supplement: S1 File — (PDF) [file pone.0241773.s003.pdf]
